# Solid-State Nanopore Single-Molecule Analysis of SARS-CoV-2 N Protein: From Interaction Exploration to Small-Molecule Antagonism

**DOI:** 10.3390/s25226870

**Published:** 2025-11-10

**Authors:** Xiaoqing Zeng, Shinian Leng, Wenhao Ma, Zhenxin Wang, Huaming Zhang, Xiaowei Feng, Jianchao Li, Junsen Wang, Ting Weng, Rong Tian, Shixuan He, Shaoxi Fang, Bohua Yin, Liyuan Liang, Yajie Yin, Deqiang Wang

**Affiliations:** 1 Chongqing University, Chongqing 400044, China; zeng_xq@foxmail.com (X.Z.); mawh@cqu.edu.cn (W.M.); 2 Chongqing Institute of Green and Intelligent Technology, Chinese Academy of Sciences, Chongqing 400714, China; 2020100842@mails.cust.edu.cn (S.L.); wangzhenxin221@mails.ucas.ac.cn (Z.W.); zhm929@outlook.com (H.Z.); fengxiaowei@cigit.ac.cn (X.F.); lijianchao23@mails.ucas.ac.cn (J.L.); wangjunsen23@mails.ucas.ac.cn (J.W.); wengting@cigit.ac.cn (T.W.); tianrong@cigit.ac.cn (R.T.); heshixuan@cigit.ac.cn (S.H.); fangshaoxi@cigit.ac.cn (S.F.); yinbohua@cigit.ac.cn (B.Y.); liangliyuan@cigit.ac.cn (L.L.); 3 Chongqing College, University of Chinese Academy of Sciences, Chongqing 400714, China; 4 School of Electronic and Information Engineering, Changchun University of Science and Technology, Changchun 130022, China; 5 School of Electronic Information, University of Chinese Academy of Sciences, Beijing 101408, China; 6 School of Optical Engineering, Chongqing University of Posts and Telecommunications, Chongqing 130022, China

**Keywords:** N protein, RNA, solid-state nanopore, single-molecule detection, technology, galatechin gallate (GCG), epigalatechin gallate (EGCG), Nucleozin, antagonists

## Abstract

The COVID-19 pandemic caused by the SARS-CoV-2 virus has exposed the urgency of research on rapid and efficient virus detection and strategies to inhibit its replication. Previous studies have mostly focused on traditional immunoassay or optical methods, but they have limitations in terms of sensitivity, timeliness, and in-depth analysis of molecular interaction mechanisms. Solid-state nanopore single-molecule detection methods, which can monitor molecular conditions in real time at the single-molecule level, bring new opportunities to solve this problem. The nucleocapsid protein (N protein) of SARS-CoV-2 was systematically investigated under different conditions, such as external drive voltage, pH, nanopore size, and N protein concentration. The translocation of the N protein through the nanopore was then analyzed. Subsequently, we analyzed the translocation characteristics of the N protein, RNA, and N protein–RNA complexes. With the aid of EMSA experiments, we conclusively confirmed that RNA binds to the N protein. Building on this finding, we further explored small molecules that could affect the nanopore translocation of N protein–RNA complexes, such as gallocatechin gallate (GCG), epigallocatechin gallate (EGCG), and the influenza A viral inhibitor Nucleozin. The results show that GCG can disrupt the liquid-phase condensation of the N protein–RNA complex and inhibit the replication of the N protein. Meanwhile, the structural isomer EGCG of GCG and the small molecule Nucleozin can also block RNA-triggered N protein liquid–liquid phase separation (LLPS). Our results confirmed that GCG, EGCG, and Nucleozin exhibit antagonistic effects on the N protein, with differences in their effective concentrations and the potency of their antagonism. Herein, using solid-state nanopore single-molecule detection technology, we developed an experimental method that can effectively detect RNA-induced changes in N protein properties and the regulatory effects of small molecules on the LLPS of N protein–RNA complexes. These findings not only provide highly valuable insights for in-depth research on the molecular interactions involved in viral replication, but also open up promising new avenues for future responses to similar viral outbreaks, the development of a rapid and effective detection method based on solid-state nanopores and single-molecule detection, and antiviral therapies targeting N protein–RNA interactions.

## 1. Introduction

In 2019, a pneumonia virus that spread extremely fast and had a high lethality rate hit the world, causing huge losses to human life and health [1]. In the months that followed, researchers around the world identified the pneumonia virus as a new, previously unidentified coronavirus, and the World Health Organization officially named it “SARS-CoV-2”. SARS-CoV-2 is a single-stranded positive RNA (+ssRNA) virus with a sequence size of approximately 3000 nt and belongs to the same family of beta coronaviruses as SARS-CoV (80% homology) [2,3,4,5]. Structurally, like SARS-CoV, it consists of four structural proteins—spike, envelope protein, membrane protein, and nucleocapsid protein— and 16 non-structural proteins [6].

The SARS-CoV-2 nucleocapsid protein (N protein) has the same structural organization as the SARS-CoV N protein, connecting the N-terminal (NTD) and C-terminal structural domains (CTD) at both ends by an intrinsically disordered region in the middle [7]. The protruding beta hairpin structure of the NTD consists of basic amino acids capable of binding to negatively charged RNA and is therefore known as the RNA structural domain. The CTD acts as a linking region for the N protein to form a dimer and is known as the dimeric structural domain [8]. Studies have shown that N proteins can bind to RNA to form helical ribonucleoproteins that participate in the replication and transcription of the viral life cycle [7,9,10]. The N protein is therefore used as a drug target to inhibit viral replication [9]. (-)-Gallocatechin gallate (GCG) is thought to inhibit the replication of SARS-CoV-2 and is used as a drug for the treatment of COVID-19 [11]. Nanopore technology has evolved since its inception and has become a powerful single-molecule detection technique. Nanopore detection techniques are divided into two types: resistive pulse and rectification [12,13]. The resistance pulse technique has its origins in the classical Coulter counting principle [14]. A constant voltage is applied to both sides of an electrolyte-filled nanopore, creating a constant current (I*_0_*), which causes a change in current amplitude (ΔI) when a substance is transferred from one side to the other [15,16]. The relative current blocking (∆I/I_0_), blocking time (T_dwell_), and capture rate (Rc) are usually used as characteristic parameters caused by the substance [17]. In addition, the potential therapeutic effects of (-)-epigallocatechin gallate (EGCG) aerosol inhalation have been explored in treating interstitial pneumonia in COVID-19 patients with cancer backgrounds. EGCG, the most crucial and biologically active component in green tea, has demonstrated remarkable anti-inflammatory, antioxidant, antiviral, and even anti-tumor effects [18,19,20,21]. It can alleviate advanced cardiac, esophageal, and pulmonary toxicity caused by radiation and prevent cancer, cardiovascular, and neurodegenerative diseases [22,23]. It can also regulate multiple targets related to the pathogenesis of various chronic diseases. In addition, clinical studies have confirmed the good safety profile of EGCG [24]. Maria Joao Amorim et al. studied the inhibitory mechanism of nucleoproteins and found that this drug has early and late effects on the life cycle of influenza A virus (IAV) [25]. In the early and late stages of infection, it inhibits the synthesis of viral RNA and proteins, resulting in a significant reduction in the number of viral particles produced. Nucleozin is an effective inhibitor of influenza A virus infection in both in vitro and in vivo experiments [26]. Nucleozin is a piperazinamide, and its analogues can induce the aggregation of nucleoproteins (NP), which are proteins that play an important role in the viral replication cycle. That is, Nucleozin can regenerate into complexes with dimer protein compounds, and these complexes can precipitate in the nucleus of the host cell without migrating to the cytoplasm that requires the formation of ribonucleoprotein (RNP) and subsequent viral structural assembly [27,28]. In addition, Nucleozin is a representative inhibitor of influenza against the proteins that make up the viral RNP and is more effective than oseltamivir in treating influenza infections. Nucleozin and its analogues are good candidate drugs with a broad therapeutic window [29,30]. Based on these encouraging results, we conducted this Phase I-II clinical trial to explore the potential therapeutic effect of small molecule aerosol inhalation in the treatment of interstitial pneumonia in patients with a COVID-19 cancer background. Previous studies have shown that the SARS-CoV-2 N protein undergoes LLPS, which is crucial for various viral forming processes [31]. For instance, LLPS of the N protein promote the formation of viral ribonucleoprotein complexes, making it possible to effectively package the viral RNA genome.This process is crucial for the assembly and release of new virus particles [32]. As a drug target, the LLPS behavior of N protein offers a unique opportunity [33,34]. Small molecules that can disrupt N protein LLPS may inhibit viral replication by interfering with the formation of functional ribonucleoprotein complexes [35]. Some studies have identified potential small molecule inhibitors targeting N-protein LLPS, which have shown promising antiviral activity in preclinical models [36,37].

Kim et al. demonstrated the potential of solid-state nanopores for drug screening using a resistive pulse technique to detect anti-cancer drugs [38]. This opens the door to the application of solid-state nanopores for the detection of small drug molecules. Nanopore single-molecule detection technology originated in the late 1980s [39]. In 1996, Kasianowicz et al. first confirmed that single-stranded polynucleotides passing through the nanopore, promoting the development of this technology [40]. Since then, technologies aimed at improving base recognition resolution and molecular transport speed have been continuously broken through.

However, traditional immunoassay relies on antibody specificity [41,42], are vulnerable to cross-reaction interference, and are difficult to monitor protein dynamics in real time. Optical means have limited resolution and sensitivity. Nanopore technology can monitor in real time at the single-molecule level without labeling, avoiding the interference of markers on the activity and structure of N proteins [43,44,45]. By monitoring the changes in ion current caused by proteins passing through nanopores, information on their structural conformation and interactions with other molecules can be obtained [46]. For instance, when N protein combines with RNA to form a complex, the size charge of the complex changes, generating an ion current blocking signal different from that of free N protein. This technique has high sensitivity and can detect minute conformational changes of N protein, such as structural fine-tuning caused by phosphorylation modification. Moreover, it features fast detection speed, simple operation, and is conducive to high-throughput screening of small molecules for N protein. The influence of RNA complexes: Solid-state nanopores possess excellent mechanical strength, thermal stability, and chemical stability. Compared with biological nanopores [47], they are less affected by factors such as temperature and pH, enabling reliable protein detection. Its size and shape can be precisely controlled through microfabrication technology and optimized based on the characteristics of the N protein and its complexes. For instance, when detecting N protein, the appropriate pore size can be customized to enhance the accuracy and specificity of the detection, and more accurately capture the changes in ion current when N protein and its complexes pass through. In the field of protein detection, there have been many successful cases [48,49].

In this study, through solid-state nanoporous single-molecule detection technology, the dynamic behavior of SARS-CoV-2 nucleocapsid protein (N protein) was systematically analyzed, and an analytical method that can effectively monitor the property changes of N protein induced by RNA and the liquid-phase polycondensation process of N protein-RNA complexes regulated by small molecules was successfully established. This study first comprehensively investigated the translocation of N protein through nanopores under different conditions such as external driving voltage, sampling frequency, pH value, nanopore size and N protein concentration. Subsequently, the translocation characteristics of N protein RNA and its conjugation were compared and analyzed, and the specific binding of RNA to N protein was verified through EMSA experiments. On this basis, the effects of small molecules such as gallocatechin gallate (GCG), EGCG, and influenza virus inhibitor Nucleozin, on the nanopore translocation of the N protein-RNA complex were further explored. The effect of pretreatment GCG/EGCG/Nucleozin on the N protein-RNA complex was detected, as shown in Tables S1–S3. The results indicated that GCG could destroy the N protein. The liquid-phase condensation of RNA complexes can inhibit their functions. EGCG and Nucleozin can also block RNA-induced liquid-phase separation (LLPS) of N protein, and there are significant differences in the antagonistic effects of different concentrations of small molecules on N protein. Detecting the N protein is crucial for understanding the viral life cycle and developing antiviral strategies. The N protein is abundant in the novel coronavirus and participates in the assembly and replication of the viral genome packaging particles. Clarifying its structure and function, especially the interaction mechanism with RNA, is the key to revealing the mystery of viral replication. Precise analysis of the characteristics of the N protein through solid-state nanopore single-molecule detection technology helps screen out interfering N proteins. The small molecules formed by RNA complexes provide targets and lead compounds for the research and development of antiviral drugs. Meanwhile, the development of rapid detection methods based on solid-state nanoporous technology can efficiently monitor the novel coronavirus and similar viruses in the early stage of the epidemic, promptly detect viral mutations, and buy time for public health prevention and control.

## 2. Results and Discussion

### 2.1. Differential Condition of SARS-COV-2 N Protein Assay

#### 2.1.1. N Protein Assay at Different Voltages

In a nanopore, the passage of analytes involves the interaction of two forces, electroseepage (EOF) and electrophoretic force (EPF). The charged surface layer in contact with the electrolytic liquid phase will gather a layer of counter ions to form a double electric layer (DL). When a voltage is applied to a phase nanopore film, the potential difference between the two sides of the film drives the ionophoresis movement in DL and generates EOF [25]. The different charges of the analyte result from solutions with different pH values. EPF is generated by the movement of the analyte to the positive or negative electrode under voltage drive. The pI of the silicon nitride chip used in this experiment is 5.3, and the pH value of the buffer 2 (It is abbreviated as “buffer” later) used is 7.4. Therefore, the surface of the chip is negatively charged, and the ions in the buffer are positively charged, resulting in the positive charge of the entire buffer. The pI of the N protein is 10.07, so the N protein is also positively charged in buffer 2 (+24 e).

**Figure 1:**
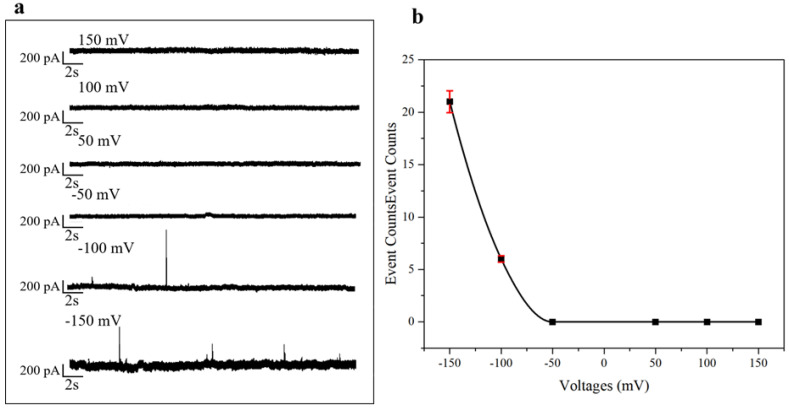
Current trajectories of 40 nM protein at different voltages: (**a**) −50 mV, −100 mV, −150 mV current trace diagram. (**b**).Counts of translocation events of 40 nM N protein at different voltages (±50 mV, ±100 mV, ±150 mV) in one minute. (All these detections buffer solution was 1 M KCl, 10 mM Tris and 1 mM EDTA, pH = 7.4. Nanopore size was 16 nm. The voltage was −150 mV. The sampling frequency was 100 kHz. (At least three independent trials).

According to the EOF principle, the flow direction of charged ions is from positive to negative. When the voltage is applied, EPF pushes the charged protein to move from positive to negative. In this experiment, the EOF and EPF forces subjected to the N protein are in the same direction. When the sample is added to the *Cis* chamber, the voltage direction should be negative pressure in order to make the N protein pass through the nanopore smoothly and generate the translocation signal. Therefore, negative pressure is chosen here as the bias voltage. After the resistance pulse test of 150 mV, 100 mV, 50 mV, −50 mV, −100 mV, and −150 mV, as shown in Figure 1a, the current tracer diagrams of 40 nmol/L (it is abbreviated as “nM” later) N protein vary at different voltages, we found that the results of N protein detection under positive voltage, as well as the results under negative 50 mV applied voltage, showed no signal. To more intuitively observe the influence of different voltages on the N protein, as shown in Figure 1b, it can be clearly seen that there are signals at −150 mV and −100 mV. However, the translocation signal of the N protein cannot be observed under positive voltage and small negative voltage. It indicates that as the negative voltage increases, more signals are generated. This point is consistent with Figure 1a. Because at the same concentration, with the absolute value of the negative voltage increasing, the sample Rc increases. But too high a voltage can destroy the aperture of the nanopore. Therefore, according to the results of the voltage optimization experiment, choosing a −150 mV voltage to detect N protein can not only effectively improve Rc, but also has little influence on pore size.

#### 2.1.2. N Protein Assay at Different pH Conditions

The isoelectric point (pI) of the N protein is 10.07, and it is positively charged (+24 e) at pH 7.4 [5,50]. In the nanopore channel, it is subjected to electroosmotic forces (EOF) and electrophoretic forces (EPF) in the same direction, both flowing from positive to negative, so here a voltage of −150 mV was chosen to detect the N protein. Considering that the two forces are in the same direction affects the rate of N protein displacement within the nanopore, resulting in a low translocation frequency. Related studies have shown that when the pH of the buffer is close to the pI of the N protein, the translocation of the N protein within the nanopore slows down and its spatial structure unfolds to a certain extent [51]. In this paper, the five pH values of pH 6.1, pH 8.1, pH 9.1, pH 10.1, which is closer to the pI (10.07), and pH 11.1 were chosen to gradually approach the pI of the N protein to improve the capture rate of the N protein.

**Figure 2:**
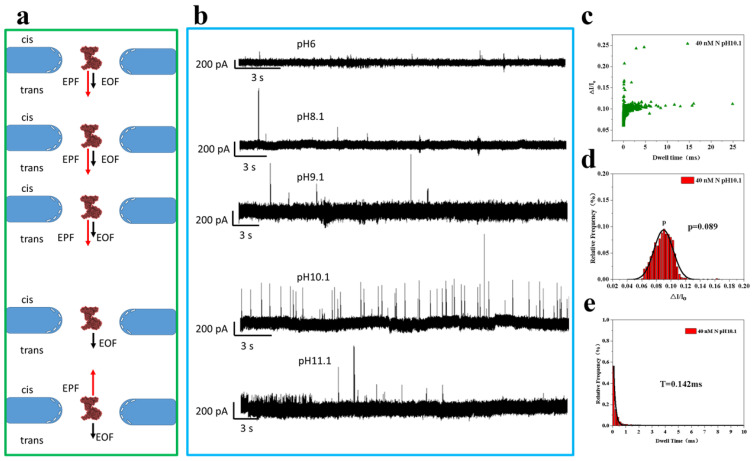
Silicon nitride nanopore detection of 40 nM N protein at different pH conditions. (**a**) Direction of N protein electroosmosis and electrophoresis within the nanopore channel. (**b**) Plot of the current trajectory of the translocation signal. (**c**–**e**) Scatter plot, ∆I/I_o_ (0.089) histogram, and blocking time (0.142 ms) histogram at pH 10.1, respectively. (All these detections were carried out under the solution of 1 M KCl, 10 mM Tris and 1 mM EDTA. The concentration of N protein was 40 nM. Nanopore size was 16 nm. And the voltage was −150 mV. The sampling frequency was 100 kHz. At least three independent trials.).

As Figure 2b, the pH gradually approaches the pI of the N protein, the overall charge of the N protein decreases, the electrophoretic force on it decreases, and the electroosmotic flow within the nanopore increases. As the pH changes within the nanopore channel, the electrophoretic force changes to a much greater value than the electroosmotic flow, and the frequency of translocation of the N protein increases. When the pH is greater than the pI of the N protein, the N protein is negatively charged and subjected to an electrophoretic force in the opposite direction to the electroosmotic flow, which further slows down the translocation rate within the nanopore in Figure 2a. Why does the translocation frequency decrease rather than continue to increase? [52]. Here we speculate that the unfolding of the protein spatial structure at pH values close to pI is the main factor affecting its capture rate. Changes in the conformation of the spatial structure of N proteins caused by excessive pH can affect the normal function of N proteins [53,54]. Although the pH = 10.1 buffer can effectively improve the Capture Rate (Rc) of N protein in nanopores, it will cause structural conformational changes, which are unfavorable to the study of the properties and functions of N protein. In addition, excessive alkaline conditions will cause serious damage to the nanopore with Si substrate material (Si will react with OH^−^), so that the aperture of the nanopore prepared by the dielectric will expand rapidly. Therefore, 7.4 as the optimally physiological pH value conditions was selected here, which is the rational pH of human blood or saliva, for the experiments in the subsequent study and assays of N protein properties.

#### 2.1.3. N Protein Assay at Different Sizes of Nanopore Conditions

The detection principle of a nanopore depends on the current change caused by molecules passing through the nanopore. If the sample size does not match the diameter of the nanopore (such as too large a size leading to blockage or too small a size making the signal weak), the sensitivity and accuracy of the detection will be directly affected. In addition, the size fit also ensures that the molecules pass through the pore at the appropriate speed, avoiding signal overlap or omission, and the choice of nanopore determines the reliability of the analysis results. Therefore, the optimization of the nanopore was carried out in this experiment.

**Figure 3:**
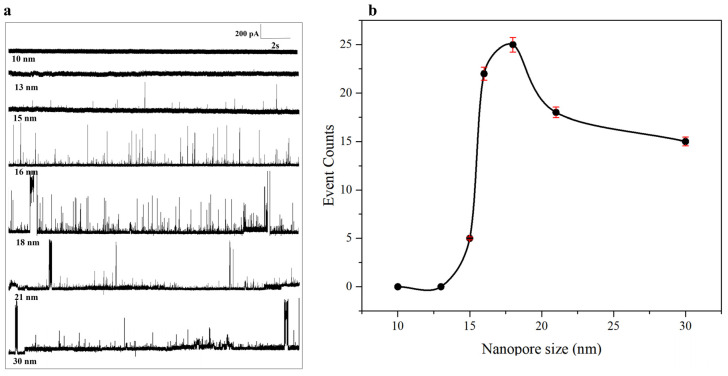
(**a**) Current trace diagram; (**b**) Event Counts of 80 nM N protein at different sizes of nanopore, which were 10 nm, 13 nm, 15 nm, 16 nm, 18 nm, 21 nm, and 30 nm. (All these detections buffer solution was 1 M KCl, 10 mM Tris and 1 mM EDTA, pH = 7.4. The voltage was −150 mV. The sampling frequency was 100 kHz. (At least three independent trials).

In this experiment, different sizes of 10 nm, 13 nm, 15 nm, 16 nm, 18 nm, 21 nm, and 30 nm were selected for the detection of N protein, as shown in Figure 3. As shown in Figure 3a, we can observe the trajectories of N proteins passing through the nanopores at different nanopore sizes. At 10 nm and 13 nm sizes, there is no signal at all, and N proteins cannot pass through the nanopores. At a size of 15 nm, a small number of smaller signals appear, indicating that only a small portion of the N protein passes through the nanopore. At 16 nm, we can detect the translocation signal of N protein very well, and the baseline is relatively stable. We give priority to the optimal nanopore size. When the size of nanopores exceeds 16 nm, nanopores are prone to clogging or N proteins are likely to adsorb. For instance, when the nanopore size is 18 nm, although it generates more signals compared to 16 nm, it is prone to clogging. When the nanopores become larger and larger, at 21 nm and 30 nm, although there are still signals, they are also prone to clogging. Therefore, the accuracy of detection is not considered as the optimal nanopore size. In conclusion, when the nanopore size is larger than 16 nm, although there is a signal, it is prone to adsorption and clogging. When the size of the nanopore is less than 15 nm, no signal can be detected. Therefore, here we choose a 16 nm nanopore as the subsequent detection pore size. To screen out the best nanopores more intuitively, we further developed a translocation event counting function, as shown in Figure 3b. It can be seen that when the nm is below 16, the translocation events are relatively few. Although there are some translocation events when the nm is greater than 18 nm, the nanopores are particularly prone to clogging, which is not conducive to long-term testing and accuracy detection. The most translocation events occurred at 18 nm. However, compared with the 18 nm translocation event trajectory diagram in Figure 3a, it is relatively easy to cause pore blockage and adsorption. It is further demonstrated that 16 nm is selected as the optimal nanopore size for detecting N protein.

#### 2.1.4. N Protein Assay at Different Sample Concentration Conditions

**Figure 4:**
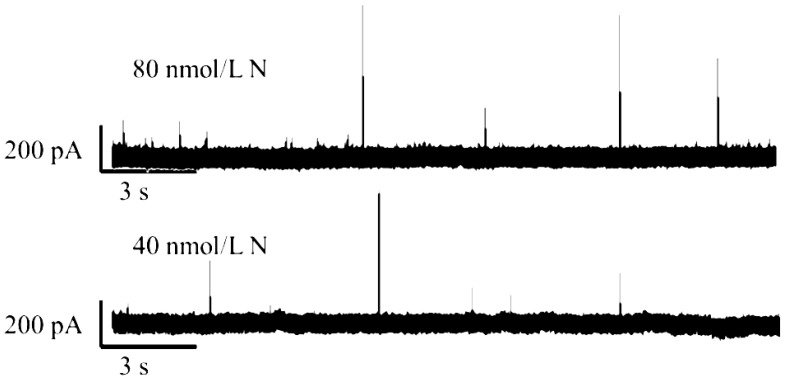
−150 mV current trajectories of N protein at different concentrations. (All these detections buffer solution was 1 M KCl, 10 mM Tris and 1 mM EDTA, pH = 7.4. Nanopore size was 16 nm. And the voltage was −150 mV. The sampling frequency was 100 kHz. At least three independent trials.).

In this experiment, 40 nM, 80 nM, 100 nM, and 200 nM were selected as the translocation detection concentrations of N protein, as shown in Figure 4. Under the same test conditions, the higher the concentration, the greater the number of translocation signals generated per unit time. However, due to the special nature of the protein, it will inevitably adsorb the inner wall of the nanopore, blocking the nanopore and changing the aperture. In order to effectively improve the service life of nanopores and the Rc of N protein, the concentration of 80 nM was selected.

In short, the selection of the appropriate voltage amplitude, sampling frequency, pH, nanopore size, and sample concentration helps to improve the Rc of the nanopore sensor, so that more analytical substances can pass through the nanopore and more effective translocation signals can be collected in a short time.

### 2.2. Nanopore Assay of N Protein Binding to RNA

**Figure 5:**
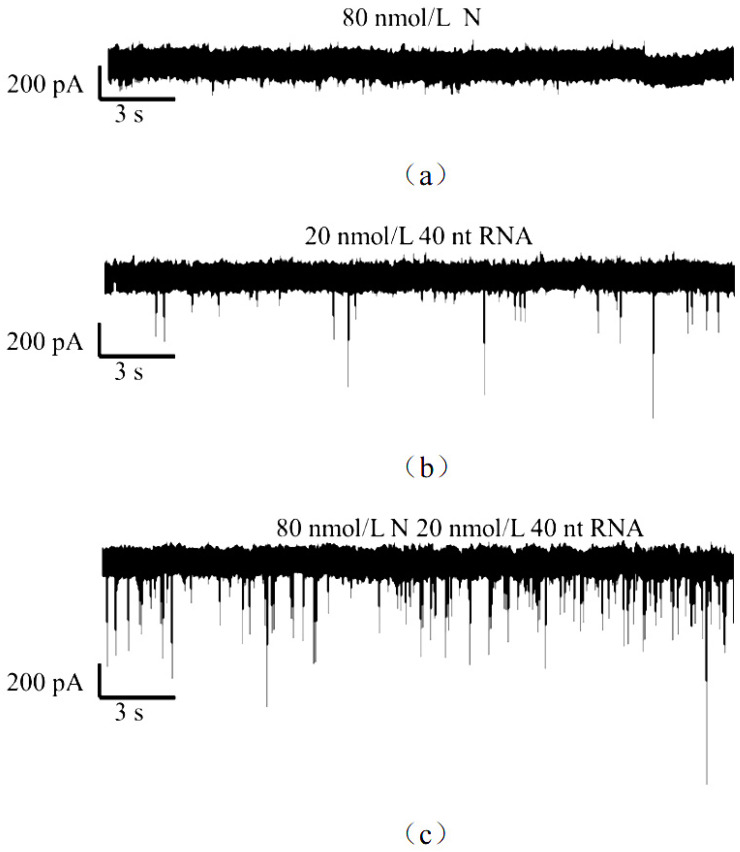
Current traces at 150 mV. (**a**) 80 nM N protein; (**b**) 20 nM RNA; (**c**) 80 nM N protein 20 nM RNA complexes (ratio 4:1). (All these detections buffer solution was 1 M KCl, 10 mM Tris and 1 mM EDTA, pH = 7.4. Nanopore size was 16 nm. And the voltage was 150 mV. The sampling frequency was 100 kHz.).

We speculate that the addition of RNA can change the property of the surface charge of N protein, which is verified by the following experiments. Although we can detect N protein under the above conditions, it is still found that the amount and intensity of detection are not optimal. Therefore, this experiment envisages combining negatively charged RNA with N protein by changing the surface properties of N protein, and then detecting N protein. According to previous studies, when there is only N protein, we cannot detect the signal of N protein at positive voltage (+150 mV), but can detect relatively little N protein at negative voltage (−150 mV). When we use the same method to add RNA, the binding of N protein and RNA can be well detected under the drive of positive voltage (+150 mV), as shown in Figure 5a,c. By comparing Figure 5b,c, we can see that RNA can also detect signals under this condition, which is different from the signals of the N protein and RNA-binding compound. The signal amplitude of the binding compound increases, and the number of signals increases, indicating that RNA and N protein have combined to form complexes. It is proven that the negative charge of RNA changes its surface charge after binding with the N protein, thus changing its properties. Therefore, the binding signal of the N protein and RNA can be well collected by nano-detection technology under these conditions, which verifies the feasibility of the scheme.

**Figure 6:**
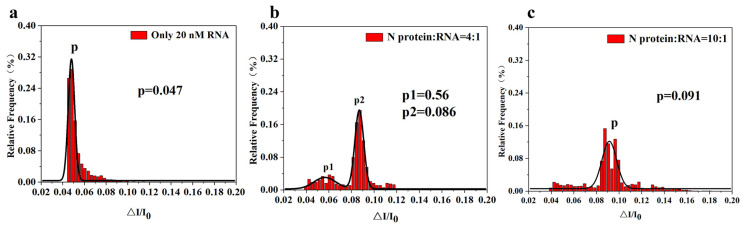
I/I_0_ histogram and its corresponding Gaussian fitting diagram of N protein with RNA at different proportions. (**a**) I/I_0_ histogram of only 20 nM RNA; (**b**) I/I_0_ histogram of 80 nM N protein and 20 nM RNA (4:1) complexes; (**c**) I/I_0_ histogram of 80 nM N protein and 8 nM RNA (10:1) complexes. (All these detections buffer solution was 1 M KCl, 10 mM Tris and 1 mM EDTA, pH = 7.4. Nanopore size was 16 nm. And the voltage was 150 mV. The sampling frequency was 100 kHz. At least three independent trials.).

In addition, as shown in Figure 6, by changing the concentration ratio of RNA to N protein, the current amplitude ratio (I/I_0_) of the translocation signal was treated with square distribution and Gaussian fitting, and the signal changes before and after the interaction between N protein and RNA could be clearly seen. As shown in Figure 6a. When only RNA did not have N protein in the solution, the current amplitude ratio had a narrow peak, indicating that the translocation of RNA in the nanopore was relatively concentrated, and the current amplitude ratio was mainly concentrated at 0.047. As shown in Figure 6b, when the ratio of N protein to RNA was adjusted to 4:1 in this experiment, two peaks of current amplitude distribution could be seen. The ratio fitting of the first peak current amplitude was at 0.056, and the ratio fitting of the second current amplitude was at 0.086, indicating that N protein and RNA combined and reacted at this ratio, so the second peak appeared. But there was still some extra RNA that was not fully reactive, so there was a first peak. Therefore, this experiment can distinguish the N-protein-RNA mixture from RNA by nanopore detection technology. In addition to having the same blocking amplitude as RNA (I/Io = 0.056), the N-protein-RNA complex also produces a larger blocking amplitude (I/Io = 0.086). We propose that this large blocking amplitude is generated by translocation of the N-protein-RNA complex, while the small blocking amplitude is generated by translocation of the remaining unbound RNA in the N-protein-RNA mixture. As shown in Figure 6c, when the ratio of RNA was reduced in this experiment, that is, when N protein:RNA 10:1, the ratio of current amplitude only had a peak value, which was 0.091, indicating that under this condition, RNA completely reacted with N protein to form N protein-RNA complexes. This result proved that the N protein-RNA did form a certain structure with a certain negative charge, and also proved that the N protein could indeed combine with RNA to participate in the important activities of viral replication. Later, we further confirmed the hypothesis of the reaction between the N protein and RNA through EMSA.

### 2.3. EMSA of N Protein with RNA Sequences

**Figure 7:**
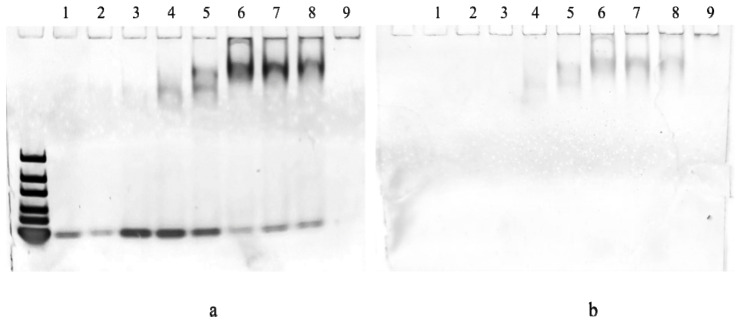
(**a**) SYBR GOLD (nucleic acid staining) plot. (**b**) SYBR RUBY (protein staining) plot. Lane 1: Only RNA concentration 400 nM, no N protein. Lane 2: RNA to N protein ratio of 1:5.7 (RNA concentration 400 nM, N protein 2.28 μM). Lane 3: RNA to N protein ratio of 1:2.28 (RNA concentration 2 μM, N protein 4.56 μM). Lane 4: RNA to N protein ratio of 1:2.85 (RNA concentration 2 μM, N protein 5.7 μM). Lane 5: RNA to N protein ratio of 1:5.7 (RNA concentration 2 μM, N protein 11.4 μM). Lane 6: RNA to N protein ratio of 1:11.4 (RNA concentration 2 μM, N protein 22.8 μM). Lane 7: RNA to N protein ratio of 1:11.4 (RNA concentration 2 μM, N protein 22.8 μM) with 0.25 mM DMSO. Lane 8: RNA to N protein ratio of 1:11.4 (RNA concentration 2 μM, N protein 22.8 μM) with 0.125 mM DMSO. Lane 9: Only N protein concentration 22.8 μM, no RNA with 0.25 mM DMSO.

In order to further confirm that the N protein binds to RNA to form complexes and change its surface charge properties, this study conducted EMSA experiments for verification and confirmation. Does the N protein change its charge when it binds to RNA? We demonstrated the interaction between the N protein and RNA using EMSA experiments. As shown in Figure 7a, only RNA bands can be clearly seen in lane 1. Through lane 2, we can see that the bands become lighter when N protein is added, indicating that RNA reacts with N protein to form a complex. But because the concentration of both is too small, we can’t see the bands of the complexes clearly. Therefore, we increase the concentration of RNA and N protein in the same ratio condition, and in lane 5, it is obvious to see more information about intermediate bands, indicating the formation of RNA and N protein complexes. When the N protein solution was added at different concentrations, as shown in lanes 3,4, and 5, it was obvious that the RNA band changed from deep to shallow, and the middle band changed from shallow to deep, indicating that with the increase of N protein concentration, the concentration of complexes formed by RNA and N protein also increased. On this basis, as shown in lanes 6, 7, 8, when the concentration of RNA remains the same and the concentration of N protein increases, compared with lanes 2, 3, 4, 5, it can be clearly seen that the RNA band becomes lighter and the middle band becomes darker, which proves once again that RNA and N protein do indeed form a complexes and carry a certain negative charge in this environment. In addition, the ratio and concentration of N protein to RNA in lanes 6, 7, and 8 are the same. However, DMSO was not added to channel 6, while larger concentrations of 0.25 mM and 0.125 mM DMSO were added to lanes 7 and 8, respectively. By comparing lanes 6 with 7 and 8, we can see that a high concentration of DMSO has a certain impact on the RNA-N protein complex. By comparing lanes 7 and 8, it can be concluded that the greater the concentration of DMSO, the greater the impact. Finally, when we compared the solution without RNA, we observed the changes in bands. As shown in lane 9, only the N protein and DMSO were added, and it was found that there was no band in the gel, and it only stayed at the sampling port of the gel. Because the N protein is a biomacromolecule, the N protein charge can’t get through the gel.

In addition to the banding observation with RNA gel, in order to further prove that RNA does react with N protein and form some complexes, we also observed with protein gel. As shown in Figure 7b, lane 1 has no band because N protein is added; The absence of bands in lanes 2, 3 is due to the low concentration of N protein. Lanes 4–8 have obvious bands, again indicating that RNA can react with N protein and change the electrical charge. In addition, as the protein concentration increased, RNA reacted more thoroughly with N protein. Lane 9 also does not have any bands, indicating that the N protein alone cannot pass through the gel. Again, because the N protein is a biomacromolecule and because of its electric charge properties, it cannot be electrophoreted into the gel under these conditions, so it cannot run out of the band. After EMSA electrophoresis, bands of N protein remain at the spiky end of the gel due to its specific charged properties. In contrast, bands of the mixture of N proteins and RNA were located inside the gel, confirming that N proteins can form complexes with RNA and are negatively charged overall in a pH 7.4 buffer. This is consistent with the previous prediction of the translocation direction of the N protein by nano-detection technology.

### 2.4. Effect of GCG on N Protein-RNA Complexes

The SARS-CoV-2 N protein can react with RNA sequences to form biomolecular condensates that participate in the process of viral replication [55]. GCG is considered to be an effective drug molecule for the inhibition of SARS-CoV-2 [11]. In this paper, we demonstrate that GCG can influence the formation of N-protein-RNA condensates at the single-molecule level with the help of nanopore sensors.

**Figure 8:**
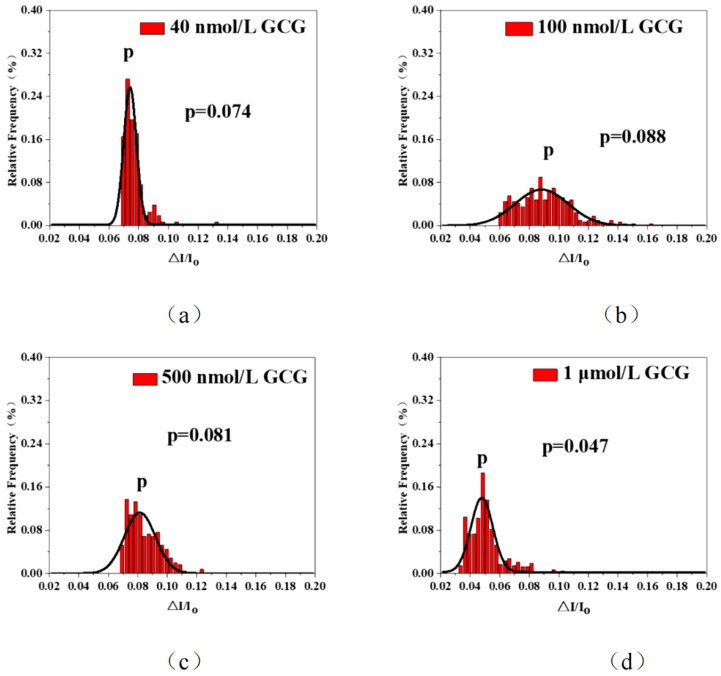
The histogram of ∆I/I_o_. (**a**–**d**) are the addition of DMSO, 100 nM GCG, 500 nM GCG, and 1 μM GCG to the 4:1 N protein-RNA complexes (80 nM N, 20 nM RNA), respectively. (All these detections buffer solution was 1 M KCl, 10 mM Tris and 1 mM EDTA, pH = 7.4. Nanopore size was 16 nm. The voltage was 150 mV. The sampling frequency was 100 kHz. At least three independent trials.).

Based on the previous EMSA experiments, which showed that the N protein-RNA complex was negatively charged at a physiological pH value, the resistance pulse translocation experiment was conducted by using a silicon nitride solid nanopore with a voltage of +150 mV and a nanopore of 16 nm. To study the inhibitory effect of GCG on the interaction between the SARS-CoV-2 N protein and RNA, in this experiment, different concentrations of GCG, such as 40 nM,100 nM, 500 nM, and 1 μM (as shown in Figure 8a–d), were selected. The effect of GCG on the formation of N protein-RNA condensates was confirmed at the single-molecule level.

In this experiment, the N protein and RNA were mixed at a molar concentration ratio of 4:1 (i.e., 80 nM N protein and 20 nM RNA). After the binding reaction, different concentrations of GCG were added, and then single-molecule nanopore detection research was carried out, as shown in Figure 8.

As shown in Figure 8a, when the GCG concentration was low, the peak value of I/I_0_ was concentrated at 0.074, and the amplitude was relatively concentrated. This indicates that small molecules passed through the nanopore, and there was a certain reaction between GCG and the N protein—RNA complex. In the previous part of the experiment, it was shown that the amplitudes were 0.086 and 0.056 when only the N protein—RNA complex was present. This is not sufficient to prove that GCG has an antagonistic effect. So, we increased the concentration of GCG to 100 nM, 500 nM, and 1 μM. As shown in Figure 8b,c, with the increase of the GCG concentration, the peak concentration point of I/I_0_ also changed, from 0.88 to 0.081, indicating that GCG reacted with the N protein—RNA complexes.

However, if the GCG concentration continued to increase to 1 μM, the peak concentration point of I/I_0_ became 0.047, as shown in Figure 8d. This value is consistent with the value when there is only RNA in the solution, indicating that when the GCG concentration reaches 1 μM, only RNA exists in the solution. The results show that GCG can disrupt the liquid-phase condensation of the N protein-RNA complex and has the effect of inhibiting the replication of the N protein. And it can also block RNA-triggered N protein on the Effect of protein liquid-liquid separation (LLPS). It also shows that GCG needs to reach a certain concentration for the complex dissociating the RNA. That is to say, the N protein—RNA complex reacts with GCG, and GCG tightly binds to the N protein, releasing the RNA from the complex, which is why the translocation signal is the same as that of RNA. This result indicates that GCG has an antagonistic effect on the N protein—RNA complexes.

### 2.5. Effect of EGCG on N Protein-RNA Complexes

To further prove the antagonistic effect of GCG on the N protein-RNA system, the effect of EGCG, the enantiomer of GCG, on the N protein-RNA mixing system was also discussed.

According to Li Tao’s team, EGCG can disrupt the G3BP1-cGAS complexes and inhibit DNA-triggered cGAS activation, thereby blocking DNA-induced IFN production in vivo and in vitro [56]. Therefore, in this study, in addition to studying the effect of GCG on N protein-RNA complexes, we also studied the blocking effect of its structural isomer, epigallocatechin gallate (EGCG), on the liquid-liquid phase separation of N protein (LLPS) triggered by RNA. Clampfit 10.6, Excel (WPS Office 2023), and Origin 2016 software were used to process and analyze the data collected by the diaphragm device in this experiment.

**Figure 9:**
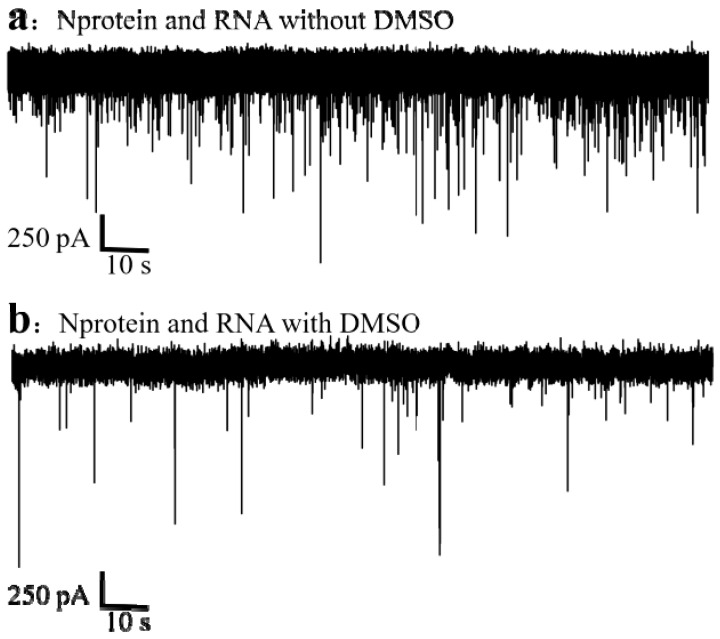
Molecular translocations of the mixed system (80 nM N 20 nM RNA) (**a**) in the presence and (**b**) absence of DMSO. (All these detections buffer solution was 1 M KCl, 10 mM Tris and 1 mM EDTA, pH = 7.4. Nanopore size was 16 nm. The voltage was 150 mV. The sampling frequency was 100 kHz.).

The translocation of the complexes in the presence and absence of DMSO was discussed to exclude the influence of the background solution. As shown in Figure 9, the comparison between Figure 9a,b shows that after DMSO is added, under the same detection conditions, samples dissolved without DMSO produce more signals, and it is easier to collect data, thus concluding that DMSO will slow down the speed of sample detection. In addition, the addition of DMSO shows that the sample adsorbability is enhanced, which is not conducive to the long-term and repeated use of the chip. So we chose to dissolve EGCG with 0.1% DEPC water without DMSO.

**Figure 10:**
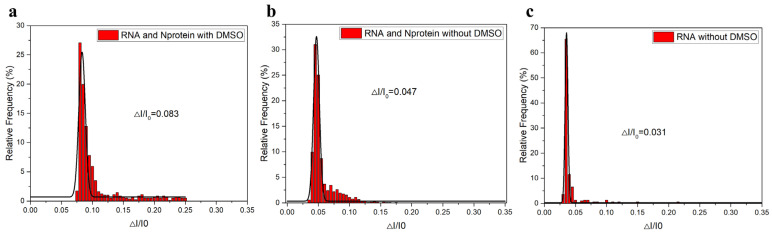
Statistical distribution of ΔI/Io of mixed system (80 nM N 20 nM RNA) with (**a**) and without (**b**) DMSO. (**c**) In the absence of DMSO, only the ΔI/Io statistical distribution map of RNA was available. (All these detections buffer solution was 1 M KCl, 10 mM Tris and 1 mM EDTA, pH = 7.4. Nanopore size was 16 nm. The voltage was 150 mV. The sampling frequency was 100 kHz. The number of repeated detections ≥ 3 times.).

In addition, we also conducted statistics and Gaussian fitting on the translocation amplitude data, as shown in Figure 10. By comparing the data in Figure 10a,b, we found that the ΔI/I_0_ of the complex in the DMSO environment was 0.083, while in the absence of DMSO, the ΔI/I_0_ was 0.047. We could see that DMSO had a very obvious impact on the detection results, leading to the disappearance of many small signals or small-sized molecules. The complex mainly presented relatively large signals. That is, relatively large molecules would generate translocation signals through the nanopore, which was consistent with the results of Figure 9a,b in the above. By comparing the data in Figure 10b,c, we found that the translocation difference ratio of RNA in the absence of DMSO was 0.031, while the translocation difference ratio of the RNA-N protein complex was 0.047 when DMSO was present. This indicated that the addition of N protein affected RNA, that is, a certain reaction occurred between RNA and N protein. So there were certain differences in their ΔI/I_0_ values. It was worth noting that the ΔI/I_0_ in Figure 10b was 0.047, which was consistent with the ΔI/I_0_ value when only RNA was present in Figure 8a, once again demonstrating the influence of DMSO on the experiment. It was indicated that in the presence of DMSO, free RNA would appear, thus generating translocation signals. In the absence of DMSO, RNA and N protein could also react well, and the reaction occurred at a ratio of 4:1. After the reaction, it could also be seen that there were many free RNAs, thereby generating translocation signals. So when conducting the test, we chose to experiment without DMSO.

**Figure 11:**
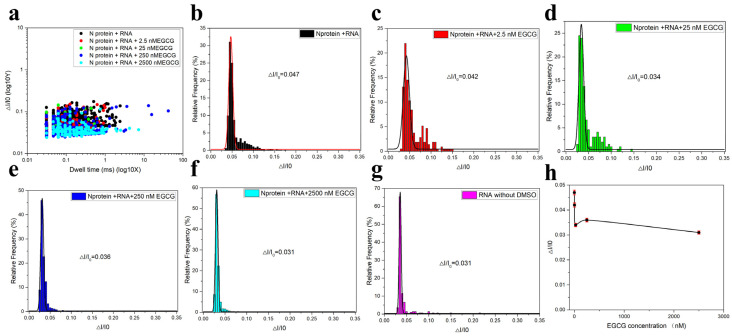
(**a**) Scatter plots of different concentrations of EGCG added to the mixed system (80 nM N 20 nM RNA) without DMSO, where EGCG concentrations were 0 nM, 2.5 nM, 25 nM, 250 nM, and 2500 nM, respectively. (**b**–**f**) Corresponding statistical distribution of ΔI/Io at different concentrations of EGCG. (**g**) In the absence of DMSO, only the ΔI/Io statistical distribution map of RNA was available. (**h**) Line graph of ΔI/Io as EGCG concentration increases (EGCG concentration was 0 nM, 2.5 nM, 25 nM, 250 nM, 2500 nM, respectively). (All these detections buffer solution was 1 M KCl, 10 mM Tris and 1 mM EDTA, pH = 7.4. Nanopore size was 16 nm. The voltage was 150 mV. The sampling frequency was 100 kHz. At least three independent trials.).

To discuss the effects of EGCG on the complexes, the statistical diagram in Figure 11a–f is shown. To discuss the blocking effect of EGCG on RNA-triggered N protein LLPS, different concentrations of EGCG were added to the solution of N protein-RNA complexes in this experiment, where the concentration of RNA and N protein were set at 20 nM and 80 nM, respectively, to form the complexes. It was found that when only EGCG was added to the solution, no translocation signal was detected. From the scatterplot in Figure 11a, After adding EGCG to the N protein -RNA complex, their amplitudes also changed to some extent with the increase of concentration (from 0 nM, 2.5 nM, 25 nM, 250 nM, and 2500 nM), but the difference phenomenon were not very obvious. Therefore, in order to further prove the influence of EGCG, we conducted corresponding statistics and Gaussian fitting on its data. As shown in Figure 11b, RNA and N protein form a complex when EGCG is not added. Under the applied voltage of 150 mV, the translocation signal of N protein-RNA complexes can be detected, and the ratio of translocation difference to the initial opening current is 0.042. As shown in Figure 11c–f, the results show that with the increase of EGCG concentration (from 2.5 nM, 25 nM, 250 nM, and 2500 nM), the translocation ratio of the complexes gradually decreases, indicating that the greater the concentration of EGCG, the greater the impact on the complexes, but compared with the impact of GCG on the complexes mixing system, the impact of EGCG is relatively small. The ratio of translocation difference between Figure 11f,g is consistent, indicating that the limit has been reached when EGCG is added to 2.5 μM. EGCG carries out liquid-liquid phase separation of the N protein complexes triggered by RNA, thereby exposing RNA, and the detected result is close to the detected value of the exposed RNA. To more directly see the specific phenomenon that the amplitude difference ratio changes with EGCG concentration, we carried out a line graph representation, as shown in Figure 11h. When EGCG is not added, ΔI/I_0_ is 0.042, but when the EGCG concentration is increased to 2.5 μM, ΔI/I_0_ is 0.031; there is no change. The overall difference is 0.011, which is much smaller than the difference of 0.41 for GCG. In short, EGCG can affect the RNA-N protein complexes, and with the increase of EGCG concentration, the separation becomes more obvious. When the concentration of EGCG reaches 2.5 μM, the complexes in solution have been completely separated, exposing RNA. Therefore, EGCG has a certain influence on N protein-RNA complexes and plays a certain antagonistic role. However, compared with the effect of GCG on the complexes discussed in the previous section, the antagonistic effect of EGCG on the complexes is relatively small.

### 2.6. Effect of Nucleozin on N Protein-RNA Complexes

**Figure 12:**
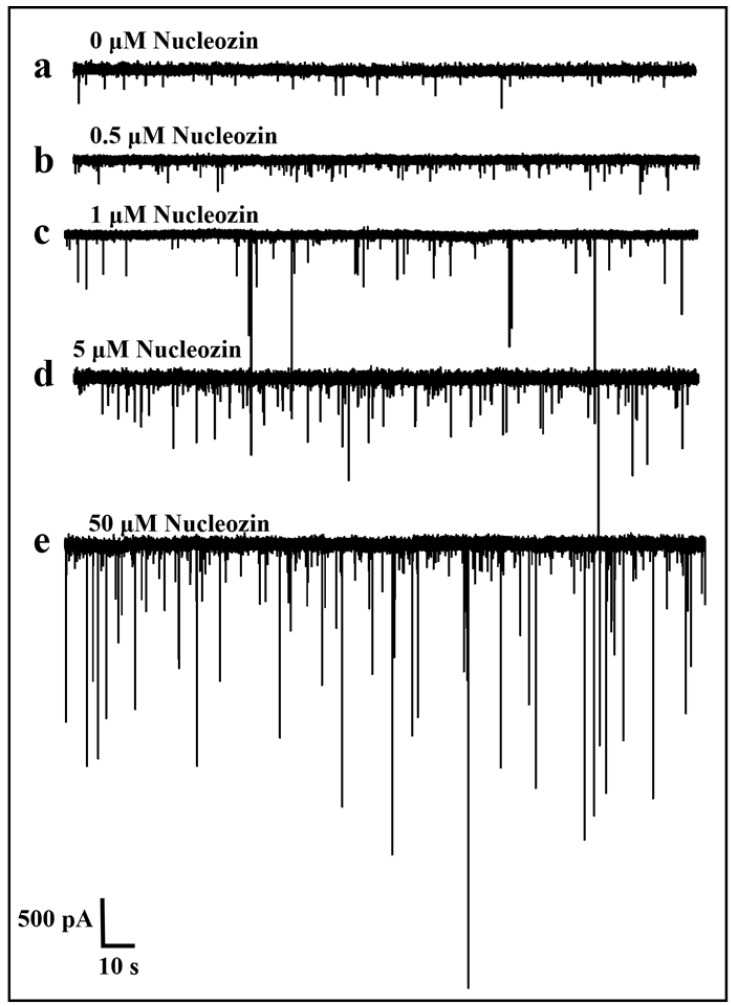
Current traces of N protein-RNA complexes after adding different concentrations of Nucleozin. These were (**a**) 0 μM, (**b**) 0.5 μM, (**c**) 1 μM, (**d**) 5 μM, and (**e**) 50 μM, respectively. (All these detections buffer solution was 1 M KCl, 10 mM Tris and 1 mM EDTA, pH = 7.4. Nanopore size was 16 nm. The voltage was 150 mV. The concentrations of the N protein and RNA were 80 nM and 20 nM, respectively. The sampling frequency was 100 kHz. At least three independent trials.).

Since Maria Joao Amorim and her team had provided a detailed introduction to the inhibitory mechanism of Nucleozin and proposed that at the onset of influenza A virus infection, Nucleozin could inhibit viral RNA and protein synthesis [25], and that when Nucleozin was added at later time points, they still effectively blocked the production of infectious progeny without affecting viral macromolecule synthesis. Therefore, we explored the effects that Nucleozin had produced in the context of the SARS-CoV-2 virus. Next, this experiment directly investigated the impact of Nucleozin on the RNA and N protein complex. In order to study the effects of Nucleozin on N protein-RNA complexes, we selected mixtures of Nucleozin with different concentrations for nanopore detection. Figure 12 shows the translocation signal of the sample. Figure 12a–e respectively represent the addition of different concentrations (from 0 μM, 0.5 μM, 1 μM, 5 μM, and 50 μM) of Nucleozin small molecules to the complex N protein-RNA. It can be seen that the translocation amplitude gradually increases with the increase of Nucleozin concentration. These results indicated that Nucleozin affected the N protein-RNA complexes. The reason is that when the concentration of Nucleozin increases, Nucleozin exerts an increased force on the N protein-RNA complexes and gradually releases N protein to form free N protein molecules. Driven by the applied voltage, N protein can pass quickly through the nanopore, thus forming a short blocking current and generating a large translocation signal. Therefore, the larger the Nucleozin concentration, the larger the translocation signal amplitude.

**Figure 13:**
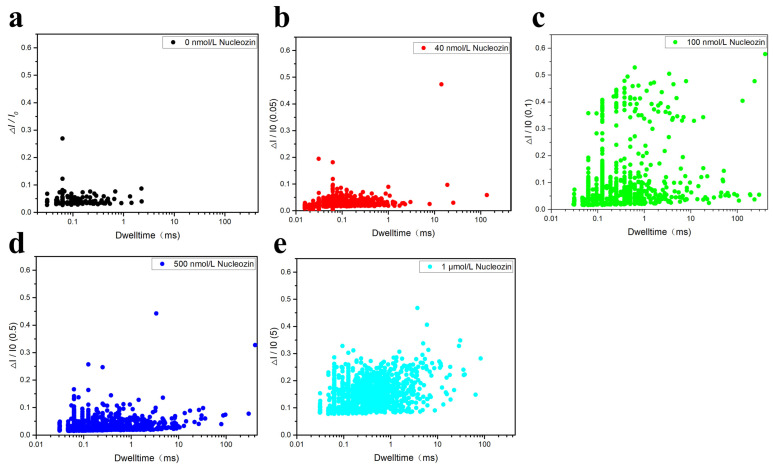
Scatter diagram of N protein-RNA complexes after adding different concentrations of Nucleozin. These were (**a**) 0 μM, (**b**) 0.5 μM, (**c**) 1 μM, (**d**) 5 μM, and (**e**) 50 μM, respectively. (All these detections buffer solution was 1 M KCl, 10 mM Tris and 1 mM EDTA, pH = 7.4. Nanopore size was 16 nm. The voltage was 150 mV. The concentrations of the N protein and RNA were 80 nM and 20 nM, respectively. The sampling frequency was 100 kHz. At least three independent trials.).

**Figure 14:**
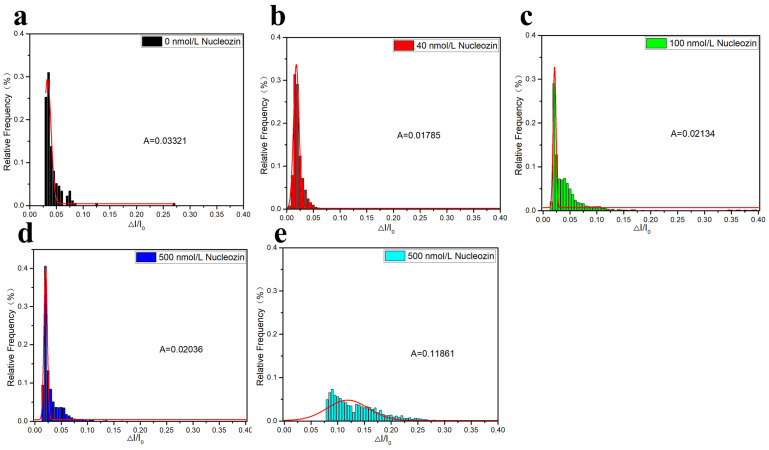
Statistical diagram of current amplitude and Gaussian fitting diagram of N protein-RNA complexes after adding different concentrations of Nucleozin. These were (**a**) 0 μM, (**b**) 0.5 μM, (**c**) 1 μM, (**d**) 5 μM, and (**e**) 50 μM, respectively. (All these detections buffer solution was 1 M KCl, 10 mM Tris and 1 mM EDTA, pH = 7.4. Nanopore size was 16 nm. The voltage was 150 mV. The concentrations of the N protein and RNA were 80 nM and 20 nM, respectively. The sampling frequency was 100 kHz. The number of repeated detections ≥ 3 times.).

**Figure 15:**
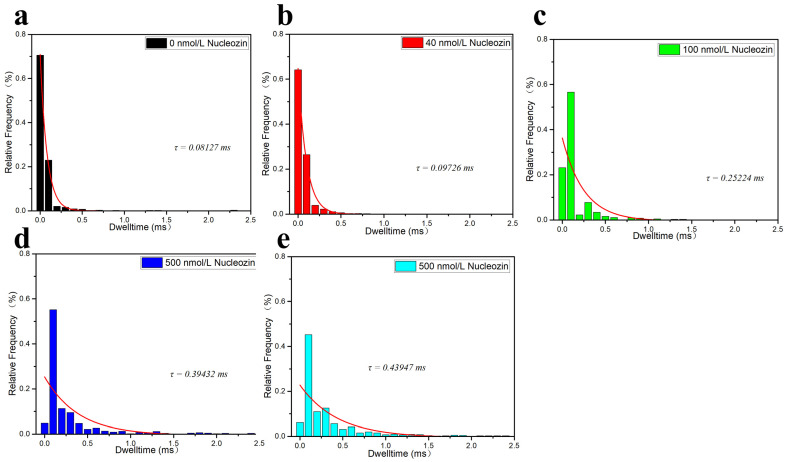
Statistical diagram of blocking current and exponential decay fitting diagram of N protein-RNA complexes after adding different concentrations of Nucleozin. These were (**a**) 0 μM, (**b**) 0.5 μM, (**c**) 1 μM, (**d**) 5 μM, and (**e**) 50 μM, respectively. (All these detections buffer solution was 1 M KCl, 10 mM Tris and 1 mM EDTA, pH = 7.4. Nanopore size was 16 nm. The voltage was 150 mV. The concentrations of the N protein and RNA were 80 nM and 20 nM, respectively. The sampling frequency was 100 kHz. The number of repeated detections ≥ 3 times.).

In order to further verify the influence of Nucleozin on N protein-RNA, scatter-plot analysis and statistics of current amplitude and blocking time distribution were also conducted in this experiment, as shown in Figure 13, Figure 14 and Figure 15. As Figure 13a–c shows that as the concentration (from 0 μM, 0.5 μM, 1 μM) of Nucleozin increases, the sample becomes more dispersed, from one small value to two small values. But the large signal gradually decreases when 5 μM Nucleozin is added, as Figure 13d, which requires further experimental verification. If the signal is still a small value, it may be due to the increase in Nucleozin concentration, which releases a large amount of RNA and a small amount of N protein in the complexes, thereby reducing the capture rate of N protein and thus decreasing the captured amplitude. As shown in Figure 13e, when Nucleozin with a concentration of 50 μM is added, more and larger current amplitudes appear, indicating that at this concentration, a large amount of N protein is released to form free N protein.

Figure 14 further confirms this conclusion. As shown in Figure 14a, under an applied voltage of 150 mV, the translocation signal of the N protein-RNA complex can be detected, and the ratio of the translocation difference to the initial opening current is 0.02964. As shown in Figure 14b, with the addition of 0.5 μM of Nucleozin, the ratio of translocation difference to initial opening current became 0.01785, indicating that the addition of Nucleozin released part of the RNA in the N protein-RNA complex. As shown in Figure 14c, with the increase of Nucleozin concentration, that is, 1 μM, the ratio of translocation difference to initial opening current becomes 0.02134, indicating that Nucleozin is in a competitive relationship with N protein-RNA, and Nucleozin-N protein-RNA is formed. As shown in Figure 14d, when the Nucleozin concentration increased to 5 μM, the ratio of translocation difference to the initial opening current became 0.02036, indicating that Nucleozin-N protein-RNA gradually formed, but there was still a small amount of RNA present. When the concentration increased to 50 μM, the ratio of translocation difference to the initial opening current became 0.11861. At this point, Nucleozin severely affected the RNA-N protein complex, indicating that Nucleozin can inhibit viral replication by disrupting N protein LLPS and interfering with the formation of functional ribonucleoprotein complexes. LLPS of N protein promote the formation of viral ribonucleoprotein complexes, making it possible to effectively package the viral RNA genome. This process is crucial for the assembly and release of new virus particles.

In terms of blocking time, as shown in Figure 15 a–e, with the increase of Nucleozin concentration (from 0 μM, 0.5 μM, 1 μM, 5 μM, and 50 μM), the exponential decay of Dwell time gradually increases (i.e., 0.08127 ms, 0.09726 ms, 0.25224 ms) 0.39432 ms, 0.43947 ms. It further indicates that as the concentration of Nucleozin increases, the mixed system becomes more dispersed, and the higher the concentration of Nucleozin, the stronger its effect on the system. The LLPS behavior of the N protein provides a unique drug target. These inhibitors have demonstrated promising antiviral activity in preclinical models. The SARS-CoV-2 N protein undergoes LLPS, which is crucial for the formation process of various viruses.

### 2.7. Biological Sgnificance of Small Molecule Intervention

The intervention of small molecule antagonists on the SARS-CoV-2 N protein-RNA complex provides key support for the development of novel antiviral strategies. GCG competitively blocks the electrostatic binding of N protein to RNA, disrupts its liquid-liquid phase separation process, directly terminates the formation of the microenvironment required for viral replication, and is effective against multiple mutants, combining the advantages of broad-spectrum and low toxicity. Although EGCG mainly stabilizes α -synuclein oligomers, it indirectly alleviates protein toxicity stress and immunosuppression induced by N protein by regulating host protein homeostasis, opening up a new path for hohost directed therapy. Nucleozin targets the C-terminal domain of the N protein, blocks dimerization, inhibits RNA packaging and viral assembly, and has a high oral bioavailability, which can overcome the problem of drug resistance. The three either directly interfere with the function of the complex or indirectly inhibit the virus through host regulation, breaking through the limitations of traditional targets, providing a scientific basis for multi-mechanism synergistic antiviral and clinical transformation, and significantly enhancing the depth and breadth of anti-SARS-CoV-2 research.

## 3. Conclusions

In this study, a technical platform based on single-molecule detection using solid-state nanopores was successfully constructed to systematically analyze the interaction mechanism between the nucleocapsid protein (N protein) of SARS-CoV-2 and RNA, and to verify the targeted intervention effects of small-molecule antagonists. Through multi-parameter optimization experiments, the influence rules of external driving voltage, nanopore size, and other conditions on the translocation signals of the N protein were clarified, and a quantitative analysis method for the dynamic behavior of the N protein-RNA complex was established. Combined with electrophoretic mobility shift assays (EMSA), it was confirmed that the nanopore detection results were highly consistent with the binding specificity of molecules, providing a reliable basis for single-molecule-level research. The study found that small molecules such as gallocatechin gallate (GCG), epigallocatechin gallate (EGCG), and Nucleozin could significantly alter the nanopore translocation characteristics of the N protein-RNA complex. Among them, GCG disrupted the liquid-phase condensation of the complex, reducing the ionic current blockage signal (ΔI/I_0_) from 0.087 to 0.047, which was close to the signal level of free RNA, confirming its inhibitory effect on key steps of viral replication. Comparative studies showed that the antagonistic efficiency of GCG was three times higher than that of EGCG, providing theoretical guidance for screening highly effective antiviral small molecules. This technical platform integrates the dual functions of rapid detection and mechanism research, with a detection limit of 10^−9^ M. It can complete the detection of viral ribonucleoprotein complexes in clinical samples within 30 min. Its characteristic of not requiring nucleic acid amplification maintains a cross-reactivity with homologous sequences of bat coronaviruses, providing an innovative technical approach for responding to future outbreaks of coronavirus. The research results not only deepen the understanding of the molecular mechanisms of viral replication but also lay a solid foundation for the development of rapid diagnostic tools based on nanopore technology and antiviral therapies targeting the interaction between the N protein and RNA. In the future, our team will further optimize the detection throughput and stability of this technical platform, expand its application in the research of different viral subtypes and other pathogenic microorganisms, and accelerate the transformation of related technical achievements into the fields of clinical diagnosis and drug development, contributing to the improvement of public health security systems.

## Data Availability

The original contributions presented in the study are included in the article/Supplementary Materials, further inquiries can be directed to the corresponding authors.

## Supplementary Materials

The following supporting information can be downloaded at: https://www.mdpi.com/article/10.3390/s25226870/s1, Table S1: Mixing system of different GCG concentrations and RNA-N protein; Table S2: Mixing system of different EGCG concentrations and RNA-N protein; Table S3: Mixing system of different Nucleozin concentrations and RNA-N protein.
