# Supplementary material for: Solid-State Nanopore Single-Molecule Analysis of SARS-CoV-2 N Protein: From Interaction Exploration to Small-Molecule Antagonism
Source: Sensors (Basel). 2025 Nov 10;25(22):6870. doi: 10.3390/s25226870 (PMC12656516; doi:10.3390/s25226870)
Supplement: Supplementary file 1 [file sensors-25-06870-s001.zip › sensors-3937308-supplementary.pdf]

Solid-State Nanopore Single-Molecule Analysis of SARS-CoV-2 N Protein: From Interaction Exploration to
Small-Molecule Antagonism

**Xiaoqing Zeng [1,2,3], Shinian Leng [2,3,4], Wenhao Ma [1,2,3], Zhenxin Wang [2,3,5], Huaming Zhang [2,3,6], Xiaowei Feng [2,3,6], Jianchao Li [2,3,5], Junsen Wang [2,3,5], Ting Weng [2,3], Rong Tian [2,3], Shixuan He [2,3], Shaoxi Fang [2,3], Bohua Yin [2,3],
Liyuan Liang [2,3], Yajie Yin [2,3,*] and Deqiang Wang [2,3,*]**

1 Chongqing University, Chongqing 400044, China; zeng_xq@foxmail.com (X.Z.); mawh@cqu.edu.cn (W.M.)
2 Chongqing Institute of Green and Intelligent Technology, Chinese Academy of Sciences,
Chongqing 400714, China; 2020100842@mails.cust.edu.cn (S.L.); wangzhenxin221@mails.ucas.ac.cn (Z.W.); zhm929@outlook.com (H.Z.); fengxiaowei@cigit.ac.cn (X.F.); lijianchao23@mails.ucas.ac.cn (J.L.); wangjunsen23@mails.ucas.ac.cn (J.W.); wengting@cigit.ac.cn (T.W.); tianrong@cigit.ac.cn (R.T.);
heshixuan@cigit.ac.cn (S.H.); fangshaoxi@cigit.ac.cn (S.F.); yinbohua@cigit.ac.cn (B.Y.);
liangliyuan@cigit.ac.cn (L.L.)
3 Chongqing College, University of Chinese Academy of Sciences, Chongqing 400714, China
4 School of Electronic and Information Engineering, Changchun University of Science and Technology, Changchun 130022, China
5 School of Electronic Information, University of Chinese Academy of Sciences, Beijing 101408, China
6 School of Optical Engineering, Chongqing University of Posts and Telecommunications,
Chongqing 130022, China
* Correspondence: yinyajie@cigit.ac.cn (Y.Y.); dqwang@cigit.ac.cn (D.W.)

1. Experimental Section

1.1 Main reagents and experimental equipment

The silicon nitride thin-film chip used in this experiment was purchased from Norcada. The thickness of the silicon nitride film was 20 nm, the substrate thickness was 200μm, and the size of the window was 10 μm × 10 μm. Yeast and pancreatic peptone were purchased from OXOID Company in the UK. AGAR and sodium chloride were purchased from the German company BioFRoxx. Tris (Hydroxymethylaminomethane), ammonium persulfate (APS), isopropanol, tetramethylethylenediamine (TEMED), glycerol, sodium imidazole sulfate (SDS), potassium chloride, boric acid, and Comas Bright Blue G-250 were purchased from Shanghai SIGMA Company. Acrylamide monomers (30%, 40%) were purchased from SOlbario. The competent cell BL21 (DE3), the chromatography column was purchased from Beijing Solaibao Technology Co., LTD. The nickel column packing material, desalination column (PD-10), was purchased from GE Company of the United States. The Bradford solution was purchased from the American company

Bole. Kanamycin sulfate was purchased from Shanghai Maclean Biochemical Technology Co., LTD. Isopropyl -β -D-thiogalactoside (IPTG) was purchased from Shanghai Biyun Tian Biotechnology Co., LTD. Methanol and Acetic acid were purchased from Chengdu Kelong Chemical Products Co., LTD. The protein molecular weight standard was purchased from Thermo Company in the United States. SYBR GOLD, trichloroacetic acid (TCA) purchased from invitrogen. (-)-Gallocatechin gallate (T3807), (-)-Epigallocatechin gallate (T2988), Nucleozin (T7330) Diethyl pyrocarbonate (T65118) was purchased from Shanghai Taoshu Biotechnology Co., LTD. Dimethyl sulfoxide (DMSO) was purchased from Shanghai SIGMA Company.

PET-28a plasmid (enzyme loci for GCTGCATATGAG CGATAACGGTCCG and AGTCTCGAGAGCCTGGGTGGAATCCGCG, the His tag is in C end), 40 nt RNA purchased from Shanghai sangon biological co., LTD.

The gene sequence of the N protein:

CATATGAGCGATAACGGTCCGCAGAACCAGCGTAACGCGCCGCGTATCACCTTTGGCG
GTCCGAGCGATAGCACCGGTAGCAACCAGAACGGCGAACGTAGCGGTGCGCGCTCTA
AACAGCGTCGTCCGCAGGGCCTGCCGAACAACACCGCTAGCTGGTTCACCGCCCTGA
CCCAGCACGGTAAAGAAGGCCTGAAATTCCCGCGTGGCCAGGGTGTTCCGATCAACA
CCAACAGCAGCCCGGATGATCAGATTGGTTATTACCGTCGTGCGACCCGTCGCATCCGT
GGTGGTGATGGCAAAATGAAAGATCTGTCTCCGCGTTGGTATTTCTACTACCTGGGCAC
CGGCCCGGAAGCAGGTCTGCCGTACGGTGCGAACAAAGATGGTATCATCTGGGTGGCG
ACCGAAGGCGCGCTGAACACCCCGAAAGACCACATCGGCACTCGTAACCCGGCGAAC
AACGCGGCTATCGTTCTGCAGCTGCCGCAGGGTACCACCCTGCCGAAAGGTTTCTACG
CAGAAGGTTCCCGTGGTGGCAGCCAGGCATCTTCACGCTCTAGCTCCCGTTCCCGCAA
CAGCAGCCGCAACAGCACCCCAGGTTCTAGCATGGGTACCTCTCCGGCGCGCATGGCG
GGTAACGGCGGTGATGCTGCGCTGGCCCTGCTGCTGCTGGATCGTCTGAACCAGCTGG
AAAGCAAAATGAGCGGCAAAGGTCAGCAGCAGCAGGGCCAGACCGTTACCAAAAAA
TCTGCGGCGGAAGCTTCCAAAAAACCGCGTCAGAAACGTACCGCAACCAAAGCGTAT
AACGTTACCCAGGCTTTCGGCCGCCGTGGTCCGGAACAGACCCAGGGCAACTTTGGT
GACCAGGAACTGATCCGTCAGGGTACCGATTACAAACACTGGCCGCAGATCGCGCAGT
TCGCACCGAGCGCGTCCGCGTTCTTCGGCATGAGCCGTATCGGCATGGAAGTTACCCC
GAGCGGCACTTGGCTGACCTACACCGGCGCGATCAAACTGGATGACAAAGATCCGAA
CTTCAAAGATCAGGTTATCCTGCTGAACAAACACATCGATGCGTACAAAACCTTCCCG
CCGACCGAACCGAAAAAAGATAAAAAGAAAAAAGCGTACGAAACCCAGGCGCTGCC
GCAGCGCCAGAAAAAACAGCAGACCGTTACCCTGCTGCCGGCGGCGGATCTGGACGA
CTTCAGCAAACAGCTGCAGCAGTCTATGTCTAGCGCGGATTCCACCCAGGCTTAAGAA
TTC. The RNA sequence: 5’-CGC GAC GUG CUCGUA CGU GGC UUU GGA GAC UCC
GUG GAGG-3’.

The Binding Buffer includes 2 mol/L NaCl, 20 mmol/L Tris, 5 mmol/L Imidazole and 0.1% TritonX-100. The Washing Buffer includes 2 mol/L NaCl, 20 mmol/L Tris, and 60 mmol/L Imidazole. Elute Buffer includes 2 mol/L NaCl, 20mmol/L Tris (pH=8.0), and 1 mol/L Imidazole. The Strip Buffer includes 0.5 mol/L NaCl, 20 mmol/L Tris (pH=8.0), and 0.5 mol/L EDTA. The Charge Buffer includes 100 mmol/L $NiSO_4$. Buffer Solution 1 includes 20 mmol/L Tris, 2 mol/L NaCl and 0.1% TritionX-100. Buffer Solution 2 includes 1 mol/L KCl, 10 mmol/L Tris and 1 mmol/L EDTA (pH=7.4). Coomassie brilliant blue is 40% Methanol, 10% Acetic acid, 50% ddH_2O and 600 mmol/L Coomassie brilliant blue. Decolorizing Solution 1 consists of 40%

volume of Methanol, 10% volume of Acetic acid and 50% volume of ddH_2O. Decolorizing solution 2 consists of 10% volume of Methanol, 7% volume of Acetic acid and 83% volume of ddH_2O. All solutions were prepared in ddH_2O with a resistance of 18.2 MΩ cm^{-1}.

The instruments used in this experiment include ultrapure water analyzer (Direct Q-3UV), ice maker (Xueke IMS-40), benchtop high-speed refrigerated centrifuge (Thermo), Luxiangyi benchtop high-speed refrigerated centrifuge (TSL-16M), ultraviolet-visible transmission and reflection spectrometer (WFH-201BJ), and microplate reader (Epoch). Bio-Tek, basic electrophoresis apparatus power supply (BIO-RAD), clean bench (SW-CJ-1FD), ultrasonic cell disruptor, ultraviolet-visible spectrophotometer (UV-5500PC), rotary mixer (BE-1100), glass cuvette (Tansoole), fixed-track shaker (TS-100) Magnetic stirrer (MS7-S), biochemical incubator (HPX-II-80), constant temperature shaker (ZWY-240), constant temperature metal bath (HB105-S2), electronic balance (JY10002), benchtop pH meter (Mettler-Toledo), Axopatch 200B diaphragm clamp amplifier.

1.2 SARS-COV-2 N protein expression and purification

For protein production, full-length N protein-protein was tagged with a C-terminal 10-Histidine tag （*pET*28a） and expressed in BL21 Escherichia coli. *E.coli* BL21 was cultured on a liquid medium with 50 µg/mL Kan antibiotics, 0.2 mM isopropyl-β-D-1-thiogalactopyranosid (IPTG) for 5 h at 37°C. After induction of expression, wet bodies were collected by centrifuge (8000 rpm,10 min, 4°C). All the steps of purification were performed at 4°C. Lysis buffer (20 mM Tris, 2 M NaCl, 0.1% TritionX-100, RNase) was used to lyse the wet bacteriophages and sonicate them. Then, the supernatant was centrifuged (13000 rpm, 1 min), and the supernatant was filtered through a 0.22 µm filter tip onto a Ni^{2+} column. Washing Buffer (20 mM Tris pH 8.0, 2 M NaCl, 60 mM imidazole) was used to remove unbound proteins, and Elute Buffer (20 mM Tris pH 8.0, 2 M NaCl, 1 M imidazole) was used to elute proteins. The eluted protein was desalted in Buffer (20 mM Tris pH 8.0, 200 mM NaCl, 1 mM EDTA, 5% glycerol) and then concentrated by ultrafiltration. The concentrated N protein SDS-Page run gel was stained with Komas Brilliant Blue to verify the Ni^{2+} purification effect.

1.3 Electrophoretic mobility shift assay (EMSA)

The EMSA experiment was performed to determine the RNA-binding ability of the N protein. Vortex 20 µM 40 nt RNA(the next text is short for RNA) with purified N protein in buffer (100 mM Tris, pH 7.4) for 10 minutes at 37℃.The samples were mixed with loading dye and then top-sampled into 8% gel and run in an ice bath at 150 mV for 30 min. The gels were stained with SYBR gold and subsequently visualized in UV light.

1.4 Nanopore Fabrication

The surface of the silicon nitride was cleaned with ethanol, ultrapure water, and isopropanol (1:1:1), followed by a clear-water treatment of the chip with a heated piranha solution. The treated

silicon nitride chip is wrapped with an O-gasket and Flow cell, filled with perforating solution (1 M KCl, TE, pH 7.4) in both chambers, and connected to the Keithley 2450 via an Ag/AgCl electrode. A controlled dielectric breakdown method was chosen for nanopore fabrication. In detail, a multi-stage current pulse method is used to achieve electrical breakdown, followed by a low voltage to expand the nanopore to the target pore size. The actual pore size is calculated with the aid of a conductivity model (Equation 1)[35]

$G=\sigma[(4L/\pi d^2)+(1/d)]^{-1}$

Where G, σ, L, and d represent the conductance of the nanopore (measured by the I-V curve), the conductivity of the electrolyte, the film thickness of the chip, and the pore size, respectively.

1.5 Detection of the sample

Flow cells fitted with nanopores are connected to the diaphragm clamp via electrodes, with the *Cis* chamber of both chambers grounded and the *Trans* chamber connected to the positive terminal. All samples were added to the *Cis* chamber in the experiment.

1.6 Data acquisition and Analysis

A patch-clamp amplifier (EPC 10 USB, HEKA) is used for ion current recording, with a sampling frequency of 100 kHz and a filter frequency of 10 kHz. All the nanopore data were acquired by the Patchmaster software. Use the ABF Utility to convert the HEKA diaphragm clamp acquisition save .dat format into an ABF format that can be read by Clampfit. Post-processing analysis of the data was carried out using Clampfit 10.6 and Origin 9.8.

1.6.1 The selection of sampling frequency and filtering frequency

In this experiment, the sampling frequency of 100 kHz and the filtering frequency of 10 kHz for I-V and BlANK were selected optimally to collect the parameter signal. The reason for the analysis is that the residence time of the protein in the nanopore is too short, and after the low-pass filter processes the generated signal, the signal amplitude is small and similar to the noise signal. Therefore, to effectively improve the Rc of the nanopore, it is necessary to increase the filtering frequency of the nanopore. Nanopore noise can be divided into (i) 1/f noise (≤100 Hz) according to its power spectral density (PSD); (ii) White noise (0.1 ~ 2 kHz); (iii) high-frequency dielectrics (1~10 kHz) and (iv) capacitors (＞10 kHz) noise. In order to avoid collecting too much noise signal and affecting the signal-to-noise ratio of the nanopore, a filtering frequency of 10 kHz is selected here. In the collection of nanopore data, overfrequency sampling (sampling frequency is 5 ~ 10 times the filter frequency) is often selected, so 100 kHz is selected as the sampling frequency of the sample, and the filtering frequency was 10 kHz.

1.7 Processing before detection for effect of GCG on N protein-RNA complexes

The apparatus for the combined system reaction was treated with 0.1% DEPC water overnight, sterilized at high temperature and dried for later use. Sterilize and dry the pipette tips used for sample preparation at high temperature and set them aside for later use. Pre-treat the purchased GCG. Dissolve the GCG powder into a sample stock solution of 5 mmol/L using DMSO and store it. In the laminar flow hood, GCG was added to the binding system of N protein and RNA, as shown in Table 1. The reaction lasted for 37 minutes for 10 minutes to obtain a mixture of GCG, N protein and RNA. Take 4 μL from the combined 10 μL system and dilute them to 200 μL with buffer 2.

Table S1 Mixing system of different GCG concentrations and RNA-N protein

RNA (20 μmol/L)	N protein (68.7 μmol/L)	100 mmol/L Tris (pH=7.4)	0.1% DEPC	DMSO
0.5μL	0.58 μL	2 μL	6.67 μL	0
0.5μL	0.58μL	2 μL	6.87μL	0.05μL
0.5μL	0.58μL	2 μL	6.82 μL	0.1μL
0.5μL	0.58μL	2 μL	6.42μL	0.5μL
0.5 μL	0.58 μL	2 μL	1.92 μL	5μL

1.8 Processing before detection for effect of EGCG on N protein-RNA complexes

Nanopore preparation: Nanopores of 16 nm were prepared for sample detection.
Sample treatment: Because Diethyl pyrocarbonate (DEPC) water can be used for the dissolution of RNA precipitation, the centrifuge tubes used in all reaction systems were cleaned overnight with 0.1% DEPC water, then sterilized at high temperature, and finally dried in an oven at 90°C. It has been explained in the previous section that although the addition of DMSO does not affect the binding of N protein to the RNA complexes, the positive charge of DMSO itself may weaken the amount of RNA charge and cause an increase in RNA Rc, thus slowing down the translocation of the complexes through the nanopore. In addition, since EGCG was soluble in polar solvents, EGCG dissolved in 0.1% DEPC water was selected for this experiment, with an initial concentration of 5 mmol/L. It was packaged in PCR tubes and stored at -80 °C to avoid instant melting and freezing, so as to prevent influence on samples. When we used it, we first took out one EGCG with the original concentration of 5 mmol/L and diluted it according to the concentration gradient of 0.5 μM, 5 μM, 50 μM, 500 μM, and 5000 μM. Then we selected sterile PCR tubes and added and mixed the samples in different proportions as shown in Table 2. After that, the mixed solution was fixed on a rotary mixer and incubated at 37°C for 10 minutes. Finally, we took out 4 μL of the well-reacted mixed solution and added it to 196 μL of the sample detection solution. In this way, the final concentrations of EGCG were 2.5 nmol/L, 25 nmol/L, 250 nmol/L, and 2500 nmol/L. During detection, the *Cis* chamber is the sample chamber, and the

ground wire (i.e., the voltage is 0 mV); the Trans terminal probe, applied voltage choice 150 mV. The gun heads used in this experiment were all sterilized at high temperature, and the additional samples were all operated on a super-clean table.

Table S2 Mixing system of different EGCG concentrations and RNA-N protein

DEPC (0.1%)	Tris (100 mmol/L pH=7.4)	N protein (15 μmol/L)	RNA (20 μmol/L)	EGCG (X mmol/L)	DMSO	Abbreviation
1 μL	2 μL	0	0.5 μL	0	0	RNA without DMSO
1 μL	2 μL	4 μL	0.5 μL	0	0	Nprotein and RNA without DMSO
1 μL	2 μL	4 μL	0.5 μL	0	2	Nprotein and RNA with DMSO
1 μL	2 μL	4 μL	0.5 μL	2.5 μL	0	2.5 nmol/L EGCG
1 μL	2 μL	4 μL	0.5 μL	2.5 μL	0	25 nmol/L EGCG
1 μL	2 μL	4 μL	0.5 μL	2.5 μL	0	250 nmol/L EGCG
1 μL	2 μL	4 μL	0.5 μL	2.5 μL	0	2500 nmol/L EGCG

1.9 Processing before detection for effect of Nucleozin on N protein-RNA complexes

Since DEPC water can be used for the dissolution of RNA precipitation, in this experiment, all the centrifuge tubes used in the reaction system were washed with 0.1% DEPC water overnight, then sterilized at high temperature, and finally dried in an oven at 90°C. Nucleozin powder was dissolved in DMSO to a concentration of 5 mmol/L, aliquoted and stored at -80 ° C. Different concentrations of Nucleozin were added to the N-protein-RNA (40nt) reaction system, and the specific sample ratios are shown in Table 5. Then, at 37°C, react for 10 minutes. Finally, take out 4 μL of the well-reacted mixed solution and add it to 196 μL of the sample detection solution. When conducting the test, the Cis terminal should be the sample terminal and grounded. The Trans terminal probe is connected, and the applied voltage is selected as 150 mV. All pipette tips used in this experiment were sterilized at high temperatures, and sample addition was carried out on a laminar flow hood.

Table S3 Mixing system of different Nucleozin concentrations and RNA-N protein

DEPC (0.1%)	Tris (100 mmol/L pH=7.4)	N protein (9.89 μmol/L)	RNA (20 μmol/L)	DMSO	Nucleozin (5 mmol/L)	Abbreviation
7.5μL	2μL	0	0.5μL	0	0	Only RNA
7.25μL	2μL	0	0.5μL	0.25μL	0	RNA&DMSO
4μL	2μL	4μL	0	0	0	Only N-protein
3.75μL	2μL	4μL	0	0.25μL	0	Nprotein&DMSO

3.25μL	2μL	4μL	0.5μL	0.25μL	0	No Nucleozin
3.45μL	2μL	4μL	0.5μL	0	0.05μL	0.5 μmol/L
3.4μL	2μL	4μL	0.5μL	0	0.1μL	1μmol/L
3μL	2μL	4μL	0.5μL	0	0.5μL	5μmol/L
0	2μL	4μL	0.5μL	0	5μL	50 μmol/L
